# Identification of the Dominant Factor for Droplet Ejection from a Tungsten Electrode during AC Tungsten Inert Gas Welding by Visualisation of Electrode Phenomena

**DOI:** 10.3390/ma16072899

**Published:** 2023-04-05

**Authors:** Kenta Iida, Hisaya Komen, Masaya Shigeta, Manabu Tanaka

**Affiliations:** 1 Joining and Welding Research Institute, Osaka University, Osaka 567-0047, Japan; h.komen@jwri.osaka-u.ac.jp (H.K.);; 2 Department of Mechanical Systems Engineering, Tohoku University, Sendai 980-8579, Japan

**Keywords:** AC TIG welding, electrode erosion, droplet ejection, splashing, temperature measurement, surface tension

## Abstract

Droplet ejections from a molten tungsten electrode during alternating current tungsten inert gas (AC TIG) welding were observed successfully by a high-speed video captured at 75,000 fps. The welding conditions and timings that were likely to occur were investigated. The electrode surface temperature was also measured. A crater was formed on the surface of the electrode, and a droplet ejection occurred following the separation of the tip of the ridge growing from the centre of the crater. A series of droplet ejections occurred on a time scale of approximately 0.4 ms. Our results showed that the high temperature of the electrode surface was the common factor for droplet ejections. The dominant force for droplet ejection was discussed by estimating the balance of forces acting on the molten electrode surface. The pressure due to surface tension was the largest pressure at any time during the AC cycle, which decreased in the second half of the EP period. Our findings suggest that the surface tension was the dominant force for changing the electrode shape and that droplet ejections occurred when the surface tension decreased due to the increase in the electrode surface temperature.

## 1. Introduction

Tungsten Inert Gas (TIG) welding is an arc welding process using tungsten as an electrode. In this welding process, arc plasma is generated between the electrode and base metal, which is the material to be welded, and the welded part is formed by melting the base metal. The formation of the welded part depends on the polarity of the electrode during this process. In electrode negative (EN) polarity, in which the electrode is the cathode and the base metal is the anode, the penetration becomes narrower and deeper and the tungsten electrode is less consumed than in electrode positive (EP) polarity. Therefore, EP polarity is rarely used by itself in TIG welding. Previous studies on EP polarity tended to remove the oxide layer covering the base metal surface by the cleaning action [1,2,3]. This is because it is difficult to ensure the required welding quality when heating the base metal without removing the oxide layer, since the layer generally has a higher melting point than the base metal and the inside of the base metal melts while the oxide layer on the surface remains unmelted. Therefore, it is effective to remove the oxide layer by the cleaning action in the EP polarity to weld metals with a high melting point oxide layer. In alternating current (AC) TIG welding, the electrode’s polarity is periodically switched between EN and EP, and this welding process is applied to weld aluminium alloys and other metals covered with an oxide layer with a high melting point because both deep penetration and the cleaning action can be obtained.

In contrast, with respect to electrode phenomena, it has been previously reported that a part of the molten electrode tip becomes detached and is ejected from the tip in the form of a droplet during AC TIG welding [4]. Since these molten electrode droplets were found to increase electrode consumption but also reduce weld quality because molten tungsten was mixed into the weld, it is required to prevent droplet ejection from the electrode. Nevertheless, there have been few quantitative studies of droplet ejection during AC TIG welding, and the mechanism of droplet ejection and the effect of welding conditions and electrode polarity on the droplet ejection have not been clarified.

Droplet ejection from an electrode is one of the most common problems to be resolved in thermal plasma processes other than welding. Peters et al. [5] showed that droplets were easily ejected from the hafnium cathode during arc ignition and arc off in an oxygen plasma cutting process. The reason for these phenomena was discussed by estimating the forces acting on the molten cathode. As a result, the electromagnetic force, the ion impact force, and the surface tension force were balanced in a steady state. In contrast, both electromagnetic and ion impact forces were found to increase due to the increase in the current density during arc ignition, whereas these forces suddenly disappeared during arc extinction, which in turn triggered an imbalance in the forces acting on the molten electrode and consequently led to droplet ejections. Hashizume et al. [6,7] investigated droplet ejection from tungsten electrodes in multiphase AC arcs, in a process where 12 tungsten electrodes were arranged radially and an AC voltage of different phases was applied to each electrode to generate numerous thermal plasmas between the electrodes. In addition, the authors focused on the fact that a part of the molten electrode was extended just before the droplets were ejected, and attempted to clarify the dominant force in droplet ejection by estimating the force acting on the ridge. As a result, droplet ejection was more likely to occur when the electrode was the cathode than when it was the anode. The diameter of the molten area measured from the observation results depended on the polarity of the electrode and was smaller in the cathodic than in the anodic period. Therefore, it was considered that droplet ejection was likely to occur in the cathodic period because of the greater electromagnetic force acting on the ridge as a result of its smaller diameter.

Because these processes and AC TIG welding differ according to the electrode materials and the characteristics of the power source used, droplet ejections in these processes are not necessarily caused by the same mechanism. In addition, this mechanism is difficult to identify because only a limited number of studies have visualised the droplet ejection process from the electrode during AC TIG welding. Therefore, the aim of the present study is to identify the dominant factors of droplet ejection during AC TIG welding in order to clarify the underlying mechanism. To achieve this aim, the droplet ejection process was visualised, and the effect of welding conditions and electrode polarity on droplet ejection was investigated. Moreover, the electrode surface temperature was measured by two-colour pyrometry.

## 2. Materials and Methods

### 2.1. Experimental Conditions

Table 1 demonstrates the welding conditions used in this study. Pure helium, which has relatively few emission line spectra and continuous spectra, was selected as the shielding gas to facilitate observations and analyses of the electrode. In addition, water-cooled copper was used as the base metal to simplify the electrode phenomena. It has been reported that when a base metal melted during arc welding, the metal vapor was generated from the base metal and transported to the electrode, which affected electrode erosion [8]. The use of water-cooled copper, which does not generate metal vapor, as the base metal is an effective method for investigating fundamental phenomena during an arc discharge and ignoring the influence of the metal vapor [9,10,11]. Here, the EP ratio represents the ratio of the EP period to one AC cycle. A water-cooled copper plate (50 mm × 50 mm × 45 mm thick) was used as the base metal. A dual-pulse TIG welder (DAIHEN (Osaka, Japan), DA300P) was used as the welding machine. Tungsten electrodes were mechanically polished before AC TIG welding, and the welding experiments were started with the electrode tips blunted.

The recent study focused on droplet ejection phenomena in a steady state. However, it was difficult to strictly determine the steady state during AC TIG welding due to the changes in the shape and temperature of the electrode with AC cycles. Therefore, a quasi-steady state was defined as the state in which a small change would occur in the shape of the molten electrode tip. Specifically, the widest diameter in the deformed region was measured at the time of the switch to EP polarity from EN polarity. Then, the quasi-steady state was determined when the rate of change was ≤1% compared to the diameter at the same time one cycle earlier.

### 2.2. Observation of an Electrode’s Appearance

**Figure 1:**
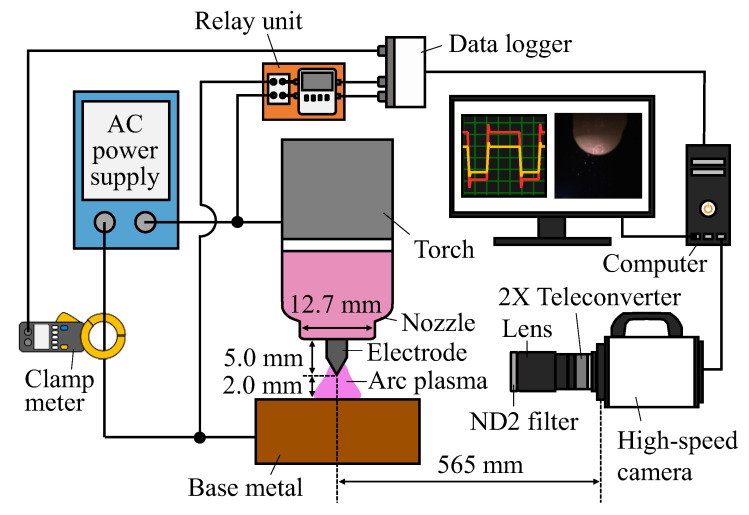
Schematic illustration of the experimental setup used to observe the electrode’s appearance.

Figure 1 shows a schematic illustration for observing an electrode’s appearance, together with the typical dimensions. A colour high-speed camera (nac Image Technology (Tokyo, Japan), MEMRECAM ACS-1 M16) was used to capture colour images of the electrode’s appearance. The lens used was composed of a single focus lens (Nikon (Tokyo, Japan), ED AF MICRO NIKKOR 200 mm 1:4 D), a teleconverter (Kenko (Tokyo, Japan), TELEPLUS HDpro 2X DGX) and a neutral density filter (Kenko, ND2). The aperture value was set to f/32, the exposure time was 0.6 μs and the frame rate was 75,000 fps. At the same time as the electrode was imaged, the voltage between the TIG torch and the base metal was measured as arc voltage using a data logger with a high-speed and high-voltage measurement unit (KEYENCE (Osaka, Japan), NR-HV04) and an interface unit (KEYENCE, NR-500). The arc voltage was measured with a delay of 0.5 s from arc ignition using a relay unit to prevent damage to the measured data by the high-frequency generated during the arc ignition. The current value during welding was measured using a clamp metre (HIOKI (Nagano, Japan), Clamp On AC/DC HiTester model 3285) and a data logger.

### 2.3. Counting Method for the Number and Timing of Droplet Ejection

**Figure 2:**
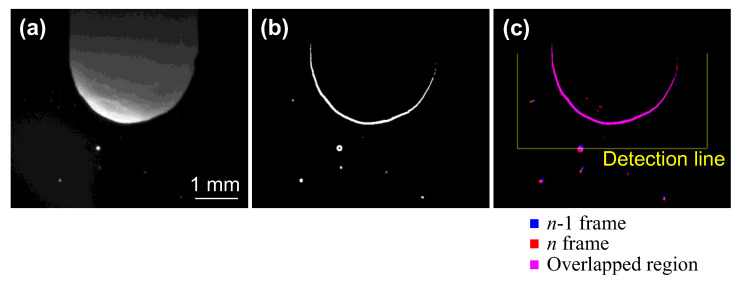
Image processing for the detection of droplet ejections: (**a**) original image of electrode’s appearance; (**b**) edge-detected and binarised image; and (**c**) superimposed image of binarised images for two consecutive frames.

The number and the timing of droplet ejection were automatically measured by the application of an image processing method to the captured images. Figure 2a depicts an example of an image obtained by a high-speed camera. As noted, the outline of the droplet was unclear because this image included light emission from the arc plasma. In Figure 2b, edge detection processing and binarisation processing using the Otsu method [12] were applied to Figure 2a to make the outline clearer. Identical droplets were identified under the following conditions: the droplet size was almost the same before and after the droplet moved, and the distance the droplet moved in 1 frame was within a distance of 10 pixels. Consequently, candidate droplets were narrowed down, and droplets with the smallest amount of movement of their weighted centre were treated as identical. Figure 2c shows the superimposed image using images at *n*−1 frames and *n* frames to which these imaging processes were applied. The blue colour in Figure 2c reflects the binarisation result for the image at the *n*−1 frame, the red colour shows the binarisation result for the image at the *n* frame and the purple colour highlights an overlapped region. When the weighted centre of a certain droplet moved across a detection line in *n*−1 and *n* frames, the droplet was counted as droplet ejection. This detection line was defined based on position of the electrode’s tip. Here, the *n* frame did not refer to the timing of detachment from the electrode but instead to the timing when the droplet crossed the detection line. Therefore, considering the droplets’ speed of movement and the distance from the electrode tip to the detection line, the timing of droplet ejection was defined as the time that was rolled back by 10 frames from *n*.

**Figure 3:**
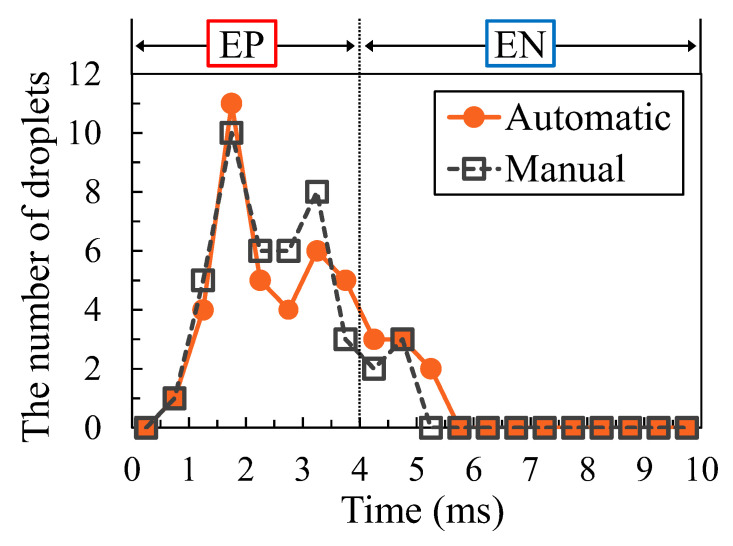
A comparison between the automatic and manual measurements.

Figure 3 demonstrates the measurement results of the number and the timing of droplet ejections using the automatic counting method to confirm the validity of this measurement method. The horizontal axis shows the time from the start of the EP period. The dotted line in this figure represents the switching time from the EP to the EN period. The plots show the number of droplets occurring during 0.5 ms, which consisted of 20 divisions of one AC cycle. A comparison between these two results revealed a distinct difference in determining the time of droplet ejection. In contrast, the timing of the peak number of droplets was consistent, as was the total number of droplets. Therefore, this automatic counting method was considered to be appropriate for identifying trends in the number and timing of droplet ejections.

### 2.4. Temperature Measurement of the Electrode Surface

Two-colour pyrometry is a method for measuring the surface temperatures of objects with unknown emissivity. The principle of this method is briefly explained here. From Planck’s radiation law, the relationship between spectral radiance L (W·m^−3^·sr^−1^), temperature T (K) and wavelength λ (m) is expressed as follows,

(1) $$L=\varepsilon \frac{2{\mathrm{hc}}^{2}}{\lambda^{5}}\frac{1}{\exp\left(\frac{\mathrm{hc}}{\lambda k_{B}T}\right)-1},$$

where ε is the emissivity, h is Planck’s constant (J·s), c is the speed of light (m·s^−1^) and $k_{B}$ is Boltzmann’s constant (J·K^−1^). When the ratio of the spectral radiances $L_{1}$ and $L_{2}$ for two different wavelengths $\lambda_{1}$ and $\lambda_{2}$ is taken, the following equation can be obtained:

(2) $$\frac{L_{1}}{L_{2}}=\frac{\varepsilon_{2}}{\varepsilon_{1}}{\left(\frac{\lambda_{1}}{\lambda_{2}}\right)}^{5}\frac{\exp\left(\frac{\mathrm{hc}}{\lambda_{1}k_{B}T}\right)-1}{\exp\left(\frac{\mathrm{hc}}{\lambda_{2}k_{B}T}\right)-1}.$$

**Figure 4:**
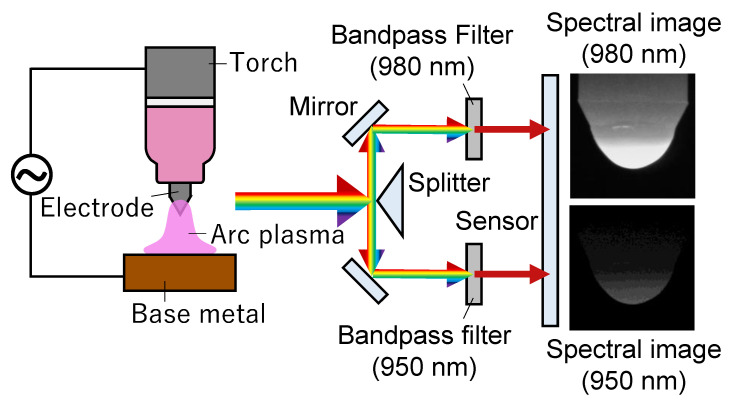
Schematic illustration of the setup for measuring electrode temperature.

Applying Wien’s approximation and assuming that the emissivity between the two wavelengths is equal, Equation (2) can be written as

(3) $$\frac{L_{1}}{L_{2}}\approx {\left(\frac{\lambda_{1}}{\lambda_{2}}\right)}^{5}\exp\left[\frac{\mathrm{hc}}{k_{B}T}\left(\frac{1}{\lambda_{1}}-\frac{1}{\lambda_{2}}\right)\right].$$

from which the temperature can be expressed as

(4) $$T=A\frac{\mathrm{hc}}{k_{B}}\left(\frac{1}{\lambda_{1}}-\frac{1}{\lambda_{2}}\right)\frac{1}{\ln\left[\frac{L_{1}}{L_{2}}{\left(\frac{\lambda_{2}}{\lambda_{1}}\right)}^{5}\right]},$$

where A is the calibration factor. In order to obtain factor A, a radiation thermometer and a tungsten lamp whose temperature was specified when a specified current value was applied to this lamp were used. Specifically, the radiant light from the tungsten lamp was measured using the two-colour pyrometry device shown in Figure 4, and the ratio of light intensities at two different wavelengths was obtained. At the same time, the temperature of the tungsten lamp was measured using the radiation thermometer. Then, the calibration factor A was obtained by correlating the ratio of light intensities obtained by the two-colour pyrometry system with the temperature obtained by the radiation thermometer. Thus, the temperature can be calculated from the ratio of the radiation intensities of two different wavelengths. Figure 4 shows a schematic illustration of the two-colour temperature measurement system used in this experiment. Using a splitter, the light incident through the objective lens was divided into two directions and then passed through bandpass filters at two different wavelengths and imaged on the CCD sensor of a high-speed camera (nac Image Technology, q1v). In this experiment, we selected 950 nm and 980 nm as the two different wavelengths.

**Figure 5:**
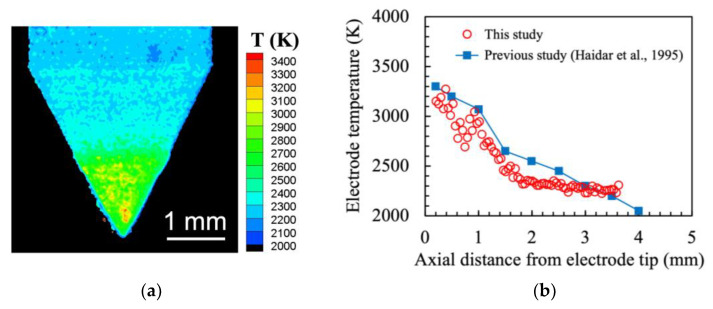
Electrode surface temperature in DCEN polarity: (**a**) two-dimensional temperature distribution; (**b**) temperature distribution along the central axis.

To verify the validity of this temperature measurement system, a TIG arc was generated on a water-cooled copper plate for 5 min using DCEN (Direct Current Electrode Negative) polarity, and the electrode temperature was measured immediately after the arc was extinguished. Figure 5a shows the two-dimensional distribution of the electrode surface temperature, while Figure 5b shows the electrode temperature distribution along the central axis from the electrode tip. The arc current was 200 A, the arc length was 5 mm, the extension was 5 mm, the shielding gas was pure argon at 5 L·min^−1^, and a 2 wt.% lanthana-doped tungsten electrode with a diameter of 3.2 mm and a tip angle of 60 degrees was used. These welding conditions were the same as in the earlier experiment conducted by Haidar et al. [13]. Although there was an error of ±10% at the maximum, the absolute values of the electrode tip temperature and trends of the temperature distribution were generally in agreement with the results of this measurement system. Therefore, it was considered that the electrode surface temperature during AC TIG welding can be measured using this method.

## 3. Results and Discussion

### 3.1. Observation Results of Electrode’s Appearance

**Figure 6:**
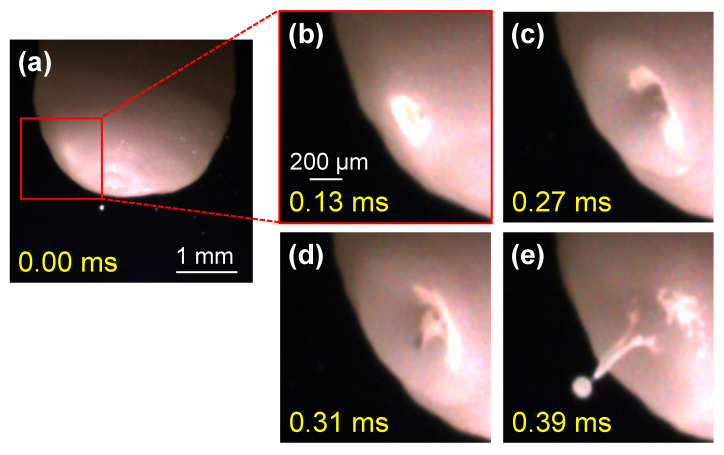
Representative images of the electrode’s appearance during AC TIG welding: (**a**) t = 0.00 ms; (**b**) t = 0.13 ms; (**c**) t = 0.27 ms; (**d**) t = 0.31 ms; and (**e**) t = 0.39 ms.

Figure 6 demonstrates an example of the observation results of the electrode’s appearance during AC TIG welding. The welding current, EP ratio and AC frequency were set to 150 A, 40% and 100 Hz, respectively. The yellow characters in this figure indicate the elapsed times from Figure 6a showing the electrode’s appearance during an EP period. As a result, irregularities or other peculiar shapes were not confirmed on the electrode surface. Subsequently, a change in the shape of the electrode surface occurred (Figure 6b), and a crater was formed (Figure 6c). Then, a ridge was formed from the centre of the crater, and droplet ejection occurred as the ridge split (Figure 6d,e). Moreover, approximately 0.4 ms elapsed from the change in the surface state to the droplet detachment, indicating that the droplet ejections from the electrode during AC TIG welding were phenomena that occurred on a timescale of approximately 0.4 ms.

### 3.2. Trajectory of the Droplet’s Ejection

**Figure 7:**
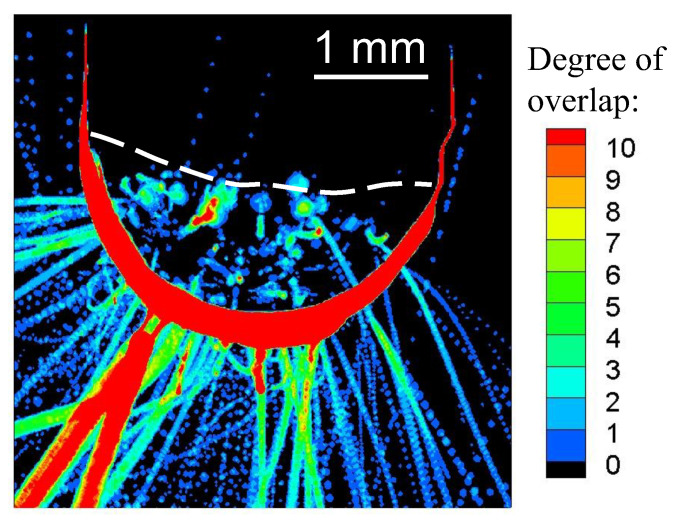
Trajectory of the droplet’s ejection during 10 AC cycles.

Figure 7 shows the results of superimposing the edge-detected and binarised images of the electrode’s appearance obtained by the camera. The welding current, EP ratio and AC frequency were set to 150 A, 40% and 100 Hz, respectively. The colours in the figure indicate the degree of overlap of the droplets’ edges and the electrode obtained by binarisation. The red colour reflects that either a larger number of droplets or droplets with slower velocity passed through. The edge of the electrode is also shown in red. By applying this image processing, information such as the number of ejected droplets, their diameters, and the trajectories of the droplets were included in a single image. The white dashed line in this figure represents the position of the melting point of tungsten, 3695 K, obtained by measuring the temperature of the electrode surface under the same welding conditions. Therefore, the solid part was above the dashed line and the liquid part was below. As shown in this figure, the droplet’s ejection occurred not only from the tip of the electrode but also from the entire molten region in the radial trajectory.

### 3.3. Measurement of the Number and Timing of Droplet Ejection

**Figure 8:**
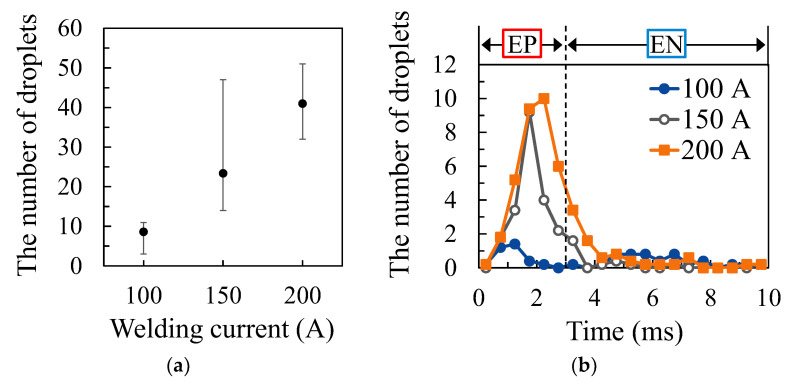
The number and timing of droplet ejection for different welding currents: (**a**) number of droplets; (**b**) timing of droplet ejections.

The number and the timing of droplet ejection were measured five times for 0.1 s at each welding condition. Figure 8a shows the result of measuring the number of droplets at each welding current. The EP ratio was set to 40%, and the AC frequency was set to 100 Hz. The plots show the average numbers of droplets occurring in 0.1 s, and the error bars show the maximum and minimum values. As shown in this graph, the number of droplets increased as the welding current increased. Figure 8b shows the results of measuring the time at which droplet ejection occurred. The plots show the average number of droplet ejections occurring in 0.1 s. The horizontal axis indicates the time that has elapsed from the start of EP polarity as 0 ms, and the dotted line in this graph indicates the timing at which the EP polarity was switched to the EN polarity. As a result, it can be seen that most of the droplet ejections occurred during the EP period. In addition, droplets were rarely ejected immediately after the start of the EP period, and droplets were ejected more easily in the latter half of this period.

**Figure 9:**
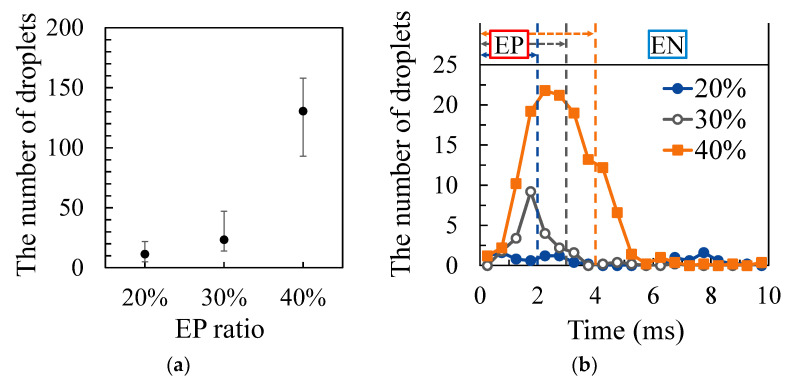
The number and timing of droplet ejection for different EP ratios: (**a**) number of droplets; (**b**) timing of droplet ejections.

Figure 9a shows the results of measuring the number of droplets for each EP ratio. The welding current was set to 150 A, and the AC frequency was set to 100 Hz. It was confirmed that the number of droplets increased as the EP ratio increased. Figure 9b shows the results of measuring the timing at which droplet ejections occurred. The blue, grey and brown dotted lines in this graph indicate the timing at which the EP polarity was switched to the EN polarity when the EP ratio was set to 20%, 30% and 40%, respectively. As a result, it was confirmed that many of the droplet ejections occurred from the middle to the latter half of the EP period.

**Figure 10:**
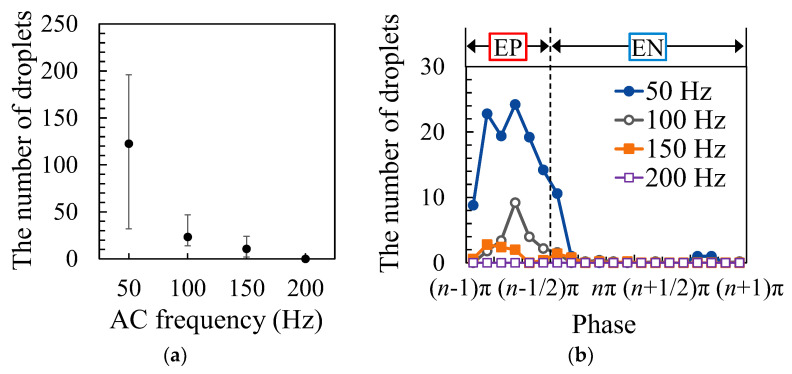
The number and timing of droplet ejection for different AC frequencies: (**a**) number of droplets; (**b**) timing of droplet ejections.

Figure 10a shows the results of measuring the number of droplets for each AC frequency. The welding current was set to 150 A, and the EP ratio was set to 30%. Similar to the previous experiment, it was confirmed that the number of droplets increased as the AC frequency decreased. Figure 10b shows the measured result of the timing of droplet ejections. However, in this case, the horizontal axis indicates the phase and not time because the time of one AC cycle differs depending on the AC frequency. From Figure 10a, it was found that droplet ejections occurred mainly during the EP period.

### 3.4. Electrode Surface Temperature

**Figure 11:**
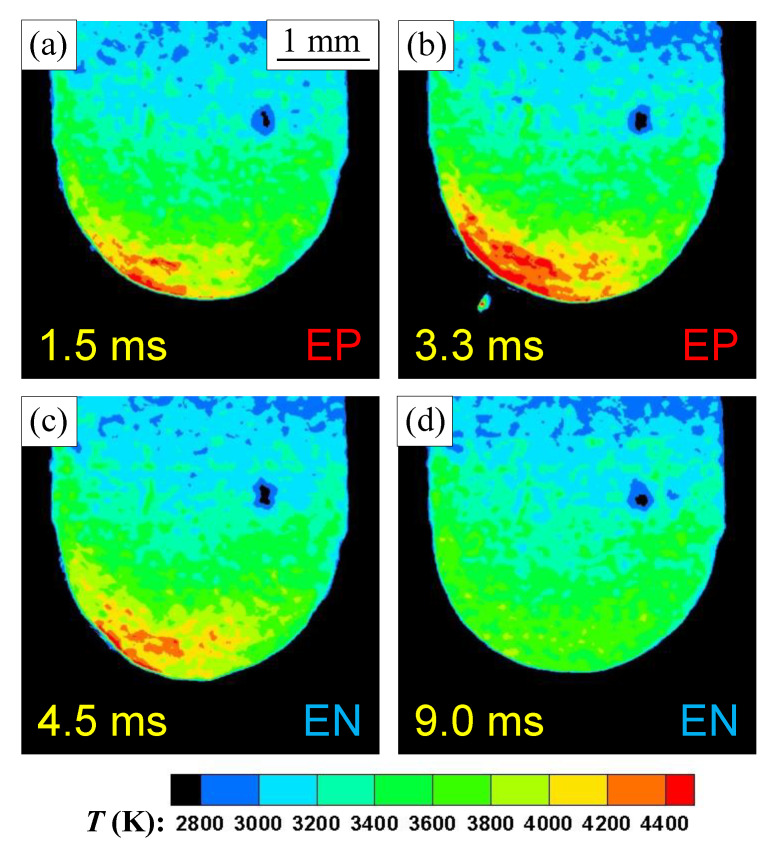
Two-dimensional distribution of the electrode surface temperature during 1 AC cycle: (**a**) t = 1.5 ms; (**b**) t = 3.3 ms; (**c**) t = 4.5 ms; and (**d**) t = 9.0 ms.

**Figure 12:**
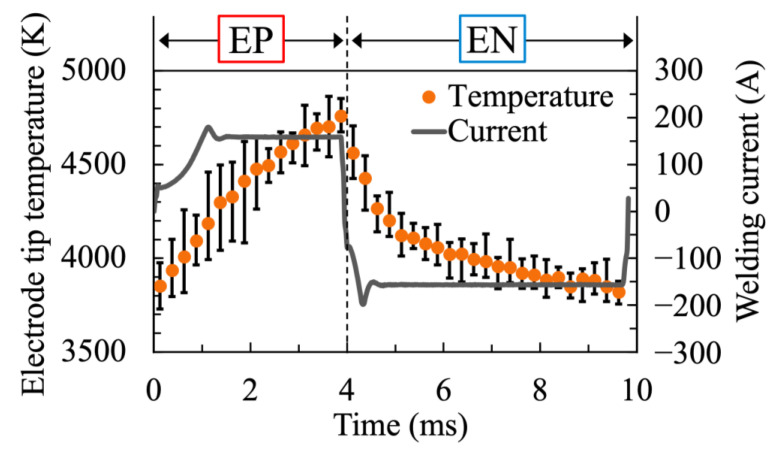
Temperature variation of the electrode tip during AC cycles.

Figure 11 shows two-dimensional distributions of the electrode surface temperature during AC TIG welding. The welding current, EP ratio and AC frequency were set to 150 A, 40% and 100 Hz, respectively. The yellow characters in this figure indicate the elapsed time from the start of EP polarity. Under these conditions, the time taken to switch from EP polarity to EN polarity was 4.0 ms. The change in the electrode’s surface temperature with the AC cycle and the droplet ejection from the high temperature area were visualised. Figure 12 demonstrates the transition of the electrode tip’s temperature during the AC cycle, together with the measured welding current waveforms. The plot shows the ensemble average over 10 AC cycles, and the error bars show the maximum and minimum values. After the switch to EN polarity, the electrode temperature continued to decrease, reaching a temperature of approximately 3800 K.

**Figure 13:**
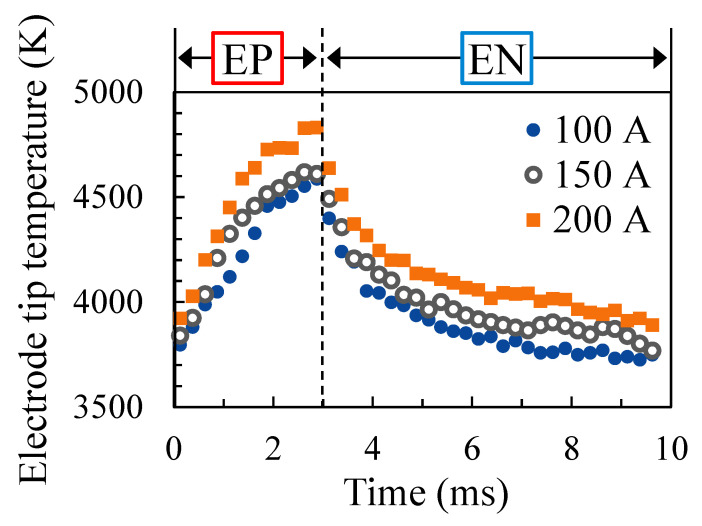
Temperature variation of the electrode tip for different welding currents.

Figure 13 shows the measured temperature variation of the electrode tip at each welding current. The EP ratio was set to 40%, and the AC frequency was set to 100 Hz. The plots show the ensemble average over 10 AC cycles, revealing that the higher the welding current the higher the electrode tip temperature at all times. For all welding currents, the electrode temperature increased during the EP period and decreased during the EN period.

**Figure 14:**
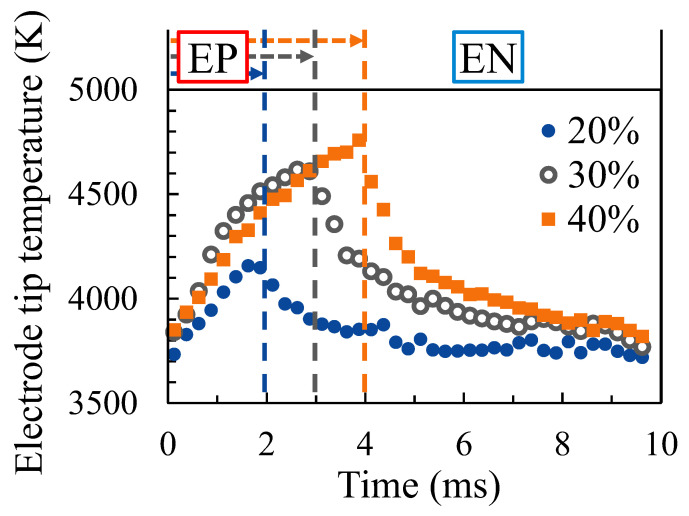
Temperature variation of the electrode tip for different EP ratios.

Furthermore, Figure 14 shows the measured temperature variation of the electrode tip at each EP ratio. The blue, grey and brown dashed lines in this figure indicate the timing of the switch from EP polarity to EN polarity when the EP ratio was set to 20%, 30% and 40%, respectively. The welding current was set to 150 A and the AC frequency was set to 100 Hz. For each EP ratio, the electrode temperature continued to increase during the EP period, while the electrode temperature continued to decrease during the EN period until the end of the EN polarity when the EP ratio was 30% and 40%. In contrast, the electrode temperature decreased for approximately 3 ms after the beginning of the EN polarity, but remained almost constant thereafter when the EP ratio was 20%.

**Figure 15:**
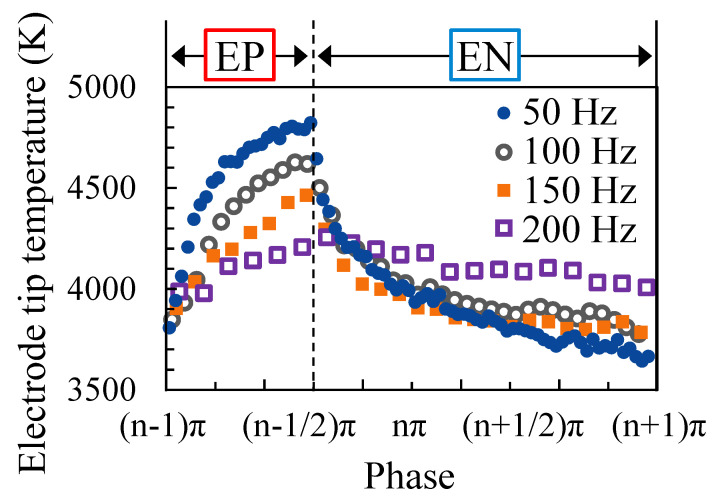
Temperature variation of the electrode tip for different AC frequencies.

Finally, Figure 15 shows the temperature of the electrode tip measured at each AC frequency. The welding current was set to 150 A and the EP ratio was set to 40%. Our findings revealed that the lower the AC frequency the larger the change in the electrode temperature with the phase change, and the higher the maximum temperature. Moreover, it was clarified that the electrode temperature just before the end of the EN polarity was lower at lower AC frequencies.

### 3.5. Common Factors When Droplet Ejection Occur

Based on the experimental results stated above, it can be suggested that the welding conditions and timing under which droplet ejection was more likely to occur were as follows:

(1) higher welding current;
(2) higher EP ratio;
(3) lower AC frequency;
(4) in the latter half of the EP period.

A high welding current will increase the current density inside the electrode, inducing an increase in the electromagnetic force, shear force due to gas flow and electrode temperature due to the increase in Joule heat. In contrast, the only effect that the increase in the EP ratio had on the electrode was an increase in the electrode temperature, mainly due to the electron inflow caused by the longer EP period. The results obtained on the temperature of the electrode surface indicated that the electrode temperature was high under all welding conditions and times when droplet ejection was likely to occur. This indicated that a high electrode temperature was a common factor when droplet ejection from the electrode was likely to occur.

### 3.6. Estimation of the Pressure Balance Acting on the Electrode Surface

This section discusses why high electrode temperatures tend to cause droplet ejection. The molten electrode surface is subjected to forces such as surface tension, electromagnetic force, ion impact force and electron impact forces [4,14]. It was hypothesised that these forces caused the detachment of droplets from the electrode. In order to identify the dominant force, these forces were converted into pressures acting on the molten electrode surface. The pressure due to surface tension $P_{\mathrm{st}}$ is expressed as

(5) $$P_{\mathrm{st}}=\frac{\sigma }{r},$$

where *σ* is the surface tension and r is the radius of curvature of the molten electrode tip. In addition, σ is given by [15]:

(6) $$\sigma =2.48-3.1\times \left(T-3695\right)\times 10^{-4}.$$

The electrode temperature T is taken from our measurements, and the pressure due to the electromagnetic force $P_{j\times B}$ is described as

(7) $$P_{j\times B}=\frac{\mu_{0}I^{2}}{4\pi S},$$

where $\mu_{0}$ and I are the magnetic permeability and the welding current, respectively. S is the area of the arc root. The pressure due to ion collision $P_{i}$ is expressed as

(8) $$P_{i}=\frac{\eta I}{S}\sqrt{\frac{2m_{i}V_{c}}{e}}.$$

**Figure 16:**
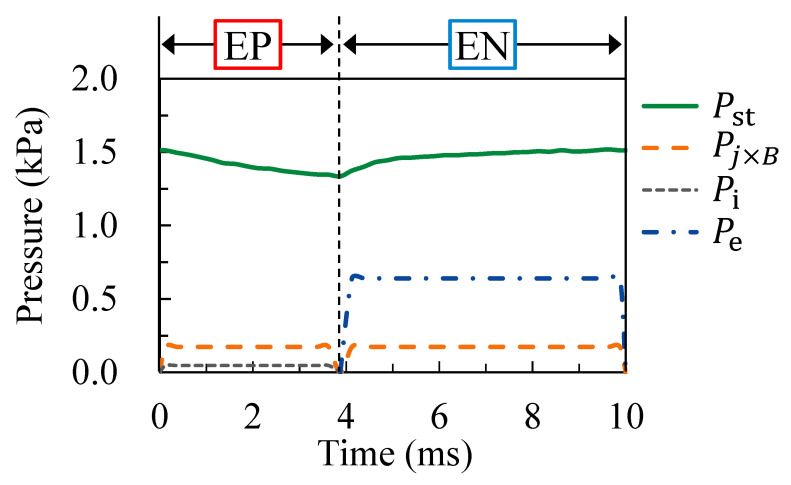
Estimated pressure balance acting on the molten electrode surface.

Here, η, $m_{i},\;\;V_{c}$ and e are the ion current fractions, the He ion mass, the sheath voltage at the cathode and the electron charge, respectively. The pressure due to the electron collision $P_{e}$ can be written as

(9) $$P_{e}=\frac{\left(1-\eta \right)I}{S}\sqrt{\frac{2m_{e}V_{a}}{e}},$$

where $m_{e}$ is the electron mass and $V_{a}$ is the sheath voltage at the anode. Table 2 shows the estimation conditions, assuming a welding current of 150 A, an EP ratio of 40% and an AC frequency of 100 Hz. Figure 16 shows the estimation result of the pressure balance acting on the molten electrode surface, suggesting that the pressure due to the surface tension was the largest pressure at any time during the AC cycle. The pressure due to the surface tension decreased by approximately 0.2 kPa in the second half of the EP period compared to the EN period and the first half of the EP period. The next largest pressures after the surface tension pressure involved an electromagnetic pressure and an ion impact pressure of approximately 0.2 kPa during the EP and 0.6 kPa during the EN periods, respectively. These results indicated that the surface tension was the dominant force in changing the electrode shape, especially in the EP period. The above results suggest that the decrease in surface tension due to the increase in electrode temperature was the dominant factor in droplet ejection.

## 4. Conclusions

In this study, droplet ejections from a molten tungsten electrode during alternating current tungsten inert gas (AC TIG) welding were observed successfully using a high-speed video captured at 75,000 fps. The welding conditions and timings that were likely to occur were investigated, and the electrode surface temperature was measured. Moreover, the dominant force for droplet ejection was discussed by estimating the balance of forces acting on the molten electrode surface. The conclusions of this paper can be summarised as follows:

(1) A crater was formed on the surface of the electrode, and droplet ejection occurred when the tip of the ridge growing from the centre of the crater was separated. A series of droplet ejections occurred on a time scale of approximately 0.4 ms.
(2) Droplet ejections were likely to occur when the electrode temperature was higher under all welding conditions and timings. In addition, the droplets were ejected from the high electrode temperature region. From these results, it can be proposed that the high temperature of the electrode surface was the common factor triggering droplet ejections.
(3) The pressure due to surface tension was the largest pressure at any time during the AC cycle, which decreased in the second half of the EP period. These results suggest that the surface tension was the dominant force for changing the electrode shape and that droplet ejection occurred when the surface tension decreased due to the increase in electrode temperature.

## Figures and Tables

**Table 1:** Welding conditions.

Welding Parameter	Value
Welding current	100, 150, 200 (A)
EP ratio	20, 30, 40 (%)
AC frequency	50, 100, 150, 200 (Hz)
Shielding gas	Pure helium
Gas flow rate	25 (L/min)
Nozzle inner diameter	12.7 (mm)
Electrode material	Pure tungsten
Electrode diameter	3.2 (mm)
Electrode tip angle	60 (deg.)
Electrode extension	5.0 (mm)
Arc length	2.0 (mm)
Base metal	Water-cooled copper

**Table 2:** Estimation conditions.

Estimation Parameter	Value
Welding current, I	150 (A)
Ion current fraction [16], η	0.1
Radius of curvature, r	1.6 × 10^−3^ (m)
Area of arc root, S	1.4 × 10^−5^ (m^2^)
Mass of ion [17], $m_{i}$	6.7 × 10^−27^ (kg)
Mass of electron [17], $m_{e}$	9.1 × 10^−31^ (kg)
Sheath voltage at the cathode [18], $V_{c}$	4.3 (V)
Sheath voltage at the anode [19], $V_{a}$	2.0 (V)
Electron charge, e	1.6 × 10^−19^ (C)
Magnetic permeability [17], $\mu_{0}$	1.26 × 10^−6^ (N · A^−2^)

## Data Availability

The data presented in this study are available on request from the corresponding authors.
